# The Pan American Health Organization-adapted Hanlon method for prioritization of health programs

**DOI:** 10.26633/RPSP.2019.61

**Published:** 2019-07-08

**Authors:** Bernard C.K. Choi, Rony A. Maza, Oscar J. Mujica, PAHO Strategic Plan Advisory Group, PAHO Technical Team

**Affiliations:** 1 Public Health Agency of Canada Public Health Agency of Canada OttawaOntario Canada Public Health Agency of Canada, Ottawa, Ontario, Canada.; 2 Pan American Health Organization, Regional Office of the World Health Organization for the Americas Pan American Health Organization, Regional Office of the World Health Organization for the Americas Washington, DC United States of America Pan American Health Organization, Regional Office of the World Health Organization for the Americas, Washington, DC, United States of America.; 3 Individual authors and affiliations at the end of the paper Individual authors and affiliations at the end of the paper Individual authors and affiliations at the end of the paper.; 4 Individual authors and affiliations at the end of the paper Individual authors and affiliations at the end of the paper Individual authors and affiliations at the end of the paper.

**Keywords:** Health priorities, decision making, organizational, strategic planning, Pan American Health Organization, Prioridades en salud, toma de decisiones en la organización, planificación estratégica, Organización Panamericana de la Salud, Prioridades em saúde, tomada de decisões gerenciais, planejamento estratégico, Organização Pan-Americana da Saúde

## Abstract

**Objectives.:**

To document the underlying science of how the Pan American Health Organization (PAHO) adapted the Hanlon method, which prioritizes disease control programs, to its wider range of program areas and used it to implement the PAHO Strategic Plan 2014 – 2019.

**Methods.:**

In 2014, PAHO established a Strategic Plan Advisory Group (SPAG) with representatives from 12 Member States to work closely with the PAHO Technical Team to adapt the Hanlon method to disease and non-disease control programs. Three meetings were held in 2015 – 2016 during which SPAG reviewed existing priority-setting methods, assessed the original Hanlon method and subsequent revisions, and developed the adapted method. This project was initiated by Member States, facilitated by PAHO, and conducted jointly in transparent and horizontal technical cooperation.

**Results.:**

From the original Hanlon equation, the PAHO-adapted method maintains components A (size of problem), B (seriousness of problem), and C (effectiveness of intervention), drops component D (PEARL – Propriety, Economics, Acceptability, Resources, and Legality), and adds component E (inequity) and F (institutional positioning). The PEARL score was dropped because it serves a purpose for pre-screening process, but not in the priority-setting process for PAHO.

**Conclusions.:**

The PAHO-adapted Hanlon method provides a refined approach for prioritizing public health programs that include disease and non-disease control areas. The method may be useful for the World Health Organization and country governments with similar needs.

## RESUMEN

**Objetivos.:**

Documentar los fundamentos científicos a través de los cuales la Organización Panamericana de la Salud (OPS) adaptó el método de Hanlon, que sirve para asignar prioridades a los programas de control de enfermedades, para aplicarlo al conjunto de áreas programáticas y ejecutar el Plan Estratégico de la OPS 2014-2019.

**Métodos.:**

En el 2014, la OPS creó un Grupo Asesor sobre el Plan Estratégico integrado por representantes de 12 Estados Miembros cuya misión era colaborar estrechamente con el equipo técnico de la OPS para adaptar el método de Hanlon para su aplicación a los programas de control de enfermedades y otras áreas programáticas. Se celebraron tres reuniones entre el 2015 y el 2016, en las cuales el Grupo Asesor examinó los métodos existentes de establecimiento de prioridades, evaluó el método de Hanlon original y sus revisiones, y elaboró el método adaptado. Este proyecto fue iniciado por los Estados Miembros, facilitado por la OPS y llevado a cabo conjuntamente mediante una cooperación técnica transparente y horizontal.

**Resultados.:**

El método adaptado por la OPS mantiene los componentes A (magnitud del problema), B (gravedad del problema) y C (eficacia de la intervención) de la ecuación de Hanlon original, prescinde del componente D (resumido en la sigla PEARL, en inglés: pertinencia, factibilidad económica, aceptabilidad, disponibilidad de recursos y legalidad), e incorpora los componentes E (inequidad) y F (posicionamiento institucional). La puntuación PEARL no fue incluida porque solo cumple una función en el proceso de preselección, no durante el proceso de establecimiento de prioridades en el que estaba trabajando la OPS.

**Conclusiones.:**

El método de Hanlon adaptado por la OPS proporciona un enfoque más preciso para la asignación de prioridades a los programas de salud pública relativos al control de enfermedades y a otras áreas programáticas. El método podría resultar útil para la Organización Mundial de la Salud y para aquellos gobiernos nacionales con necesidades similares.

## RESUMO

**Objetivos.:**

Documentar o processo de base científica empregado pela Organização Pan-Americana da Saúde (OPAS) para adaptar o método de Hanlon, que define a priorização de programas para controle de doenças, às diversas áreas programáticas da OPAS e descrever como esta metodologia foi usada para implementar o *Plano Estratégico da OPAS 2014-2019*.

**Métodos.:**

Em 2014, a OPAS formou um grupo consultivo sobre o Plano Estratégico, composto por representantes de 12 Estados Membros, para colaborar estreitamente com a Equipe Técnica da OPAS na adaptação da metodologia de Hanlon aos programas para controle de doenças e não doenças. Foram realizadas três reuniões em 2015–2016 em que se examinaram os métodos existentes para definição de prioridades, avaliaram-se o método de Hanlon original e versões revisadas posteriores e elaborou-se uma metodologia adaptada. Este projeto foi de iniciativa dos Estados Membros, com o auxílio da OPAS, conduzido conjuntamente com cooperação técnica transparente e equiparada.

**Resultados.:**

Partindo da equação original de Hanlon, a metodologia adaptado pela OPAS mantém os componentes A (magnitude do problema), B (gravidade do problema) e C (eficácia da intervenção), elimina o componente D (PEARL – pertinência, viabilidade econômica, aceitação, recursos e legalidade) e acrescenta os componentes E (iniquidade) e F (posicionamento institucional). A pontuação do componente PEARL foi excluída porque serve à finalidade de pré-seleção, não ao processo de definição de prioridades para a OPAS.

**Conclusões.:**

O método de Hanlon adaptado pela OPAS oferece um enfoque aprimorado para definir as prioridades programáticas em saúde pública que abrangem áreas de controle de doenças e não doenças. A metodologia pode ser útil à Organização Mundial da Saúde e aos governos de países com necessidades semelhantes.

Priority-setting is an important component of strategic planning (1). Given that “When everything is a priority, nothing is a priority” (2), strategic planning can be difficult to carry out. There are many available methods for priority-setting. Among them, the Hanlon method was first described in 1954 and was only applicable to disease control (3). It was subsequently augmented with an equation by Hanlon and Pickett that calculates a basic priority rating (BPR) score for ranking health problems (4). Organizations such as the Pan American Health Organization (PAHO) require a broader method to cover the full range of programs.

This article summarizes the findings of a research project conducted by PAHO and 12 Member States to adapt the Hanlon method for use in developing and implementing the PAHO Strategic Plan 2014 – 2019 (5). The purpose of this article is not to publish the new methodology. The PAHO-adapted Hanlon equation has already been published by the PAHO 55th Directing Council in 2016 (6). That document (6) was required immediately in order to begin applying the new equation to implementation of the Strategic Plan. Although it did not describe the science behind the new methodology, the document did recommend subsequent publication by a scientific journal to ensure the integrity and soundness of the adapted method through external peer-review. Publication of the scientific methodology would also contribute to the regional and global scientific community by highlighting the role that PAHO played in spearheading innovation in this area, and making the adapted method globally accessible. This article seeks to fulfill the recommendations, as well as provide a consolidated historical account of the various revisions of the Hanlon method since 1984, documenting its evolution in one place.

In 2013, during the development of the Strategic Plan (5), a Programmatic Priorities Stratification Framework was drafted. The framework included a stratification exercise to group all PAHO programs into one of three priority tiers (high, medium, or low). Two working groups explored using the Hanlon method to rank programs, and proposed an initial modification to consider non-disease programs and institutional positioning. Challenges in applying the modified Hanlon method were reported. For example, the method gave an unreasonably high priority ranking to disease-oriented programs, and low ranking to non-disease-oriented programs.

In 2014, a Strategic Plan Advisory Group (SPAG) was established to refine the Framework. Its purpose was to: (a) review other published priority-setting methods; (b) assess the strengths and weaknesses of the original Hanlon method and subsequent revisions; and (c) develop a PAHO-adapted Hanlon method. Given the limitations of the original Hanlon method, the objective of this study was to refine the methodology to suit the breadth of PAHO programs.

## MATERIALS AND METHODS

This research project was conducted during the development of the PAHO Strategic Plan 2014 – 2019. More than 15 priority-setting methods were reviewed in search of a reasonably objective and potentially useful method to prioritize PAHO programs (6). Several criteria for the allocation of resources had been developed by PAHO, such as size and severity of the problem associated with a given program. It was on this basis that the Hanlon method (4) was chosen as a starting point.

This study combined expert consultation by an advisory group with methodology development by a technical group. Because the final, adapted method would affect all countries and territories of the Americas, the experts needed to represent the entire Region. The expert team, the SPAG, included representatives from 12 Member States hailing from all four sub-regions of the Americas (North America, Central America, South America, and the Caribbean); namely, the Bahamas, Brazil, Canada, Chile, Costa Rica, Ecuador, El Salvador, Jamaica, Mexico, Paraguay, Peru, and the United States of America.

In order to facilitate the process, the SPAG established a technical core group of experts, led by Canada, that included several technically-oriented members of the SPAG and the PAHO Technical Team. The aim of this technical group was to review published priority-setting methods and to develop an adapted Hanlon method. This group also provided technical inputs to address issues and challenges identified by SPAG. This collaborative approach proved to be an effective way to achieve the project objectives. SPAG worked with the PAHO Technical Team via virtual and face-to-face meetings.

The technical core group presented findings to three SPAG face-to-face meetings for consideration and approval. The first meeting was held in May 2015 in Washington, D.C. It established an action plan to address Member States’ request to improve the Framework, and produced a revised PAHO-adapted Hanlon equation with refined definitions of several variables. The second meeting was held in August 2015 in Mexico City. It pilot-tested an improved version of the PAHO-adapted Hanlon method and developed a process and timeline for application of the new method by Member States. Pilot testing was conducted during the meeting by two separate groups: the 12 country representatives of the SPAG and 20 senior managers of the Mexican Secretariat of Health. The third meeting was held in April 2016 in Washington, D.C. It validated the final version of PAHO-adapted Hanlon method through a final pilot test by SPAG members.

## RESULTS

There are a wide variety of methods for priority-setting (6). Simple and often subjective methods include dotmocracy or dot democracy (7); forced rankings (8); nominal group method (4, 8); and simple voting procedure (8). More objective but time-consuming methods include the Delphi method (8) and the Hanlon method (4). There are also complex statistical methods such as multi-criteria decision analysis (9). Of the wide range of methods reviewed, it was decided that the Hanlon method was the most appropriate method for adaptation to the PAHO purpose. Some methods, such as dotmocracy and simple voting, are too simplistic, while others, such as complex statistical methods, are too demanding for computational ability. The SPAG perceived that, through innovating the equation’s components, the Hanlon method could provide the versatility required to accommodate both the technical and political considerations deemed relevant to a priority-setting process at an organization as complex as PAHO.

### Original Hanlon method and its revisions

The original Hanlon equation was published in 1984 (4). A priority score is assigned to each disease program using the equation below:

$$(BPR)=\frac{(A+B)C}{3}\times D$$

where A is the size of the problem on a scale of 0 – 10 points; B is the seriousness of the problem (0 – 20 points), calculated as the sum of four factors, each worth 0 – 10 points (urgency, severity, economic loss, and involvement of other people); C is the effectiveness of the intervention (0 – 10 points); D is its feasibility (0 or 1), determined by five factors (each 0 or 1) that are referred to as “PEARL” (propriety, economics, acceptability, resources, and legality). Because the obtainable product of the four components (A, B, C, and D) has a range of 0 – 300 points, dividing by 3 gives the BPR a range of 0 – 100.

Vilnius and Dandoy (10) made several methodological changes to the Hanlon method. To avoid the possibility of the total score of the four factors of component B (seriousness) exceeding the range of 0 – 20 points, each factor is allowed a range of 0 – 5 points instead of 0 – 10 points. They also suggest adding a benefit-cost ratio (B ÷ C) to the equation.

$$BPR=\frac{\left(A+B)(C+B÷C\right)}{6}\times D$$

where B ÷ C is benefit-cost ratio (0 – 10 points). With the five components, dividing by 6 gives the BPR a range of 0 – 100.

Neiger and colleagues (1) made a significant contribution to the Hanlon method by deleting the PEARL component:

Earlier versions of the BPR model suggested that analysis of the PEARL criterion should occur after all data had been collected and translated to BPR scores. Since the function of PEARL is to determine whether stakeholders should proceed with or eliminate a health problem, this criterion should be analyzed prior to data collection and analysis (1).

In other words, they consider PEARL to be a pre-screening process to identify the health problems for ranking and not part of the priority-setting process:

$$BPR=\frac{(A+B)C}{3}$$

### PAHO-adapted Hanlon method

The PAHO-adapted Hanlon method was designed to rank the breadth of PAHO programs, both disease-oriented and non-disease-oriented (Table 1). The original method and all revisions had been designed for ranking only disease-oriented programs. They would not work for PAHO because measures for disease and non-disease programs can go in different directions. That is, while an increase in the magnitude of a disease (e.g., tuberculosis prevalence) suggests a need for more attention (increased priority), an increase in the coverage of health systems or public health interventions (e.g., immunization coverage) suggests improved performance (not requiring increased priority). To resolve this incoherence, in the PAHO-adapted method, all programs being ranked are framed as a problem (Table 2). For example, for diseases, the “size of problem” refers to a high prevalence or incidence of morbidity or mortality, while for health systems and public health interventions, it refers to the lack of resources or a deficiency in program coverage.

The PAHO context also needs two new components: E (inequity) and F (institutional positioning):

$$BPR=\frac{(A+B+E)C}{5.25}\times F$$

**TABLE 1:** Programs by group, PAHO Strategic Plan 2014 – 2019

Group	Programs^a^
Disease-oriented programs	1.1 HIV/AIDS and STIs 1.2 Tuberculosis 1.3 Malaria and other vector-borne diseases (including dengue and Chagas) 1.4 Neglected, tropical, and zoonotic diseases 1.5 Vaccine-preventable diseases (including maintenance of polio eradication) 2.1 Noncommunicable diseases and risk factors 2.2 Mental health and psychoactive substance use disorders 2.3 Violence and injuries 2.4 Disabilities and rehabilitation 2.5 Nutrition (poor nutrition)
Non-disease-oriented programs	3.1 Women, maternal, newborn, child, adolescent, and adult health, and sexual and reproductive health 3.2 Aging and health 3.3 Gender, equity, human rights, and ethnicity 3.4 Social determinants of health 3.5 Health and the environment 4.1 Health governance and financing; national health policies, strategies, and plans 4.2 People-centered, integrated, quality health services 4.3 Access to medical products and strengthening of regulatory capacity 4.4 Health systems information and evidence 4.5 Human resources for health 5.1 Alert and response capacities (for IHR) 5.2 Epidemic- and pandemic-prone diseases 5.3 Emergency risk and crisis management 5.4 Food safety

a Abbreviations: HIV = human immunodeficiency virus; AIDS = acquired immunodeficiency syndrome; STI = sexually transmitted infection; IHR = International Health Regulations.

***Source***: Prepared by the authors from the study results.

**TABLE 2.** Turning all disease and non-disease programs into a problem, a novel technique used by the PAHO-adapted Hanlon method to include both disease and non-disease programs in priority setting

	Disease programs	Non-disease programs
Consideration	A disease is a bad thing	A health system or public health intervention is a good thing
Defining “a problem”	High “access” to a disease, and especially a serious disease	Low access to a good program
Defining “no problem”	Little or no disease	High access to a good program
Measuring “size of problem”	Size of problem refers to a high prevalence or incidence of disease	Size of problem refers to the lack of resources or coverage of the program
Measuring “seriousness of problem”	Seriousness refers to worsening problem, severity, economic loss, and negative impact due to disease	Seriousness refers to worsening problem, severity, economic loss, and negative impact due to lack of program or program deficiency
Measuring “effectiveness of intervention for problem”	Effectiveness of intervention refers to effective ways to reduce the problem (disease)	Effectiveness of intervention refers to effective ways to reduce the problem (lack of coverage or program deficiency)

***Source***: Prepared by the authors from the study results.

where A is the size of the problem (0 – 10 points), meaning prevalence or incidence of the disease (for disease-oriented programs) or extent of system or program deficiencies (for non-disease-oriented programs); B is the seriousness of problem (0 – 20 points), derived from the sum of four factors: B1 (urgency), B2 (severity of consequences), B3 (economic loss), and B4 (negative impact on others), each with 0 – 5 possible points. For non-disease-oriented programs, this also considers seriousness associated with deficiencies of the system or program and consequences of inaction; C is the effectiveness of intervention (0 – 10 points) based on the availability of cost-effective interventions to address the problem or deficiencies in systems or programs. It is the product of 2 factors: C1 (efficacy) and C2 (reach or coverage), each with a range of 0% – 100%. For non-disease-oriented programs, this is a qualitative assessment of effectiveness. E is inequity (0 – 5 points), measured by the degree of differential occurrence of disease or access to health programs between socially-determined population sub-groups (i.e., according to gender, ethnicity, income, literacy, urban/rural location, and/or other equity stratifiers) deemed to be unjust (i.e., arbitrary, unnecessary, avoidable). F is the institutional positioning (a factor from 0.67 – 1.50), and refers to the extent to which an institution, such as PAHO, is uniquely positioned to assist a Member State address a program need from its own perspective. Initially, the trial range of F was 0.50 (a halving effect) to 2.00 (a doubling effect), but during pilot testing these values were found to be too overwhelming. The maximum value was reduced from 2.00 to 1.50 and found to be satisfactory during further testing. As F is a multiplier, if the maximum is 1.50, the minimum is its reciprocal, or 0.67. A score of 1.00 means a Member State believes PAHO could maintain its technical cooperation at the current level. A score greater than 1.00 signifies that PAHO should increase its technical cooperation, while a score less than 1.00 means PAHO should decrease support because the Member State can deal with the problem or has another strategic partner assisting. Dividing by 5.25 gives the BPR a range of 0 – 100.

For component A (size of problem), various measures are used, e.g., the percent of the population exposed to the problem. For diseases, size of the problem refers to the prevalence/incidence. Points: 0 – 3 = low prevalence/incidence; 4 – 6 = medium; and 7 – 10 = high. For non-diseases (health systems and public health intervention programs), size of problem refers to the extent of the problem (e.g., system or program deficiency). It can be measured by percent of the population affected by the problem (e.g., without access to a health program), or degree of limitations in response capacity. Points: 0 – 3 = low percent of population adversely affected; 4 – 6 = medium; and 7 – 10 = high.

Component B (seriousness) has four factors. Urgency (B1) means whether a problem is worsening, stabilizing, or improving, or whether progress is made towards achieving the target, based on previous 5-year trend data. Points: 0 – 1 = problem improving or good progress; 2 – 3 = problem remains the same; and 4 – 5 = worsening. Severity of consequences (B2) is measured by the extent of premature mortality and disability, loss of quality of life, or burden to health services caused by the problem. Points: 0 – 1 = low; 2 – 3 = medium; and 4 – 5 = high. Economic loss (B3) is the social costs, both direct and indirect, associated with the problem. While factor B4, originally defined as “involvement of other people” (4), has a clear meaning for disease (especially infectious disease) programs, it requires a new definition for non-disease programs. Therefore, B4 is defined as a negative impact on others (other people and/or countries). This involves the concept of negative externality in economics: “A negative externality is a cost that is suffered by a third party as a result of an economic transaction” (11). In an economic transaction, the producer and consumer (first and second parties, respectively) may negatively impact on third parties, such as other individuals, organizations, or resources. This includes the ability of a problem to spread and cause other problems within a country, and the negative impact of one country (e.g., inaction) on other countries.

Effectiveness (C) is the degree of success of an intervention in producing a desired outcome under usual circumstances. Following the original definition (4), effectiveness is the product of efficacy (C1), the degree of success of an intervention under ideal (laboratory) conditions, and reach (C2), the percentage of population with effective access to the intervention; that is, the situation under usual circumstances. Both C1 and C2 are percentages, so their product is readily converted to a value between 0 – 10. For health systems and public health intervention programs, this can be a qualitative assessment of the effectiveness to correct deficiencies. Points: 0 – 3 = no effective intervention; 4 – 6 = somewhat effective; and 7 – 10 = highly effective.

Component D (PEARL) is deleted from the equation, consistent with the justifications provide by Neiger and colleagues (1) and the fact that all PAHO programs are considered relevant for ranking because they have been pre-screened as agreed with Member States.

Component E (inequity) is a new feature in the PAHO-adapted equation. Health inequities are defined as “observable differences in health between two or more socially-determined groups that are judged to be unjust; that is, arbitrary, unnecessary, and avoidable” (12). Inequity can affect people’s lives, their health, and the actions taken to prevent them from becoming diseased or to treat disease when it occurs. Points: 0 – 1 = no differential occurrence between sub-groups; 2 – 3 = moderate differential; and 4 – 5 = high differential.

Component F (institutional positioning) is another new feature of the PAHO-adapted method. This component was based on the concept first suggested by Musgrove (13) that highlights the importance of flexibility, as well as practical and political considerations in the prioritization process. This factor serves as a fine-tuner that allows Member States to identify where PAHO is uniquely positioned to collaborate with countries in addressing public health problems, taking into consideration the country’s capacity, and the contributions of other partners. Furthermore, it allows for political, strategic, and technical considerations. Scores of 0.67 – 0.99 mean the country has the capacity to respond to the scope of the program and PAHO could decrease its collaborative technical cooperation; a score of 1 means the country has some capacity, but PAHO should maintain its current level of technical cooperation; and scores of 1.01 – 1.5 indicate that the country has limited capacity and PAHO should increase technical cooperation.

### Illustration of the PAHO-adapted Hanlon method

Table 3 presents an example of the mean scores obtained from ratings by representatives of 12 PAHO Member States in the 2015 pilot test conducted in Mexico City. The 2016 pilot test conducted in Washington, D.C. by the same countries showed similar results. During the 2016 pilot test, an operational question was raised on whether it was better to score the items horizontally (sequentially scoring each program for all components) or vertically (sequentially scoring each component for all programs). This remains a personal choice, as about one-half of participants preferred each method.

## DISCUSSION

This PAHO project shows the advantages and effectiveness of the expert consensus approach. Three face-to-face meetings over a total of 8 days with an average of 20 experts (or 160 person-days), plus virtual meetings and individual time invested by members of the SPAG and the PAHO Technical Team, provided the basis for the development of the PAHO-adapted Hanlon method. The final product arising from this expert consensus approach would have been impossible for one expert to complete despite working for 160 days. A single expert would not have been able to identify and resolve the issues from the various sub-regions and multi-factor issues related to the wide range of PAHO programs. The expert group approach also fostered a team environment, as experts from different areas of the Region tackled a common problem together. At the conclusion of this project, the SPAG members had developed strong links that would enhance future country collaboration on PAHO endeavors. Such collaboration has been considered instrumental to developing new strategic frameworks for the Region, as shown by the involvement of more Member States on the new SPAG for the PAHO Strategic Plan 2020 – 2025. Expert consensus is a methodology that has been used successfully in other PAHO prioritization exercises (14).

**TABLE 3.** An example to illustrate the PAHO-adapted Hanlon method, based on the mean scores of components A to F provided by representatives of 12 PAHO Member States in a pilot test conducted in Mexico City, August 2015

Group	Program	Component A (Size of problem)	Component B (Seriousness of problem)	Component C (Effectiveness of intervention)	Component E (Inequity)	Component F (Institutional positioning)	Basic Priority Rating	Ranking
Score range		0 – 10	0 – 20	0 – 10	0 – 5	0.67 – 1.5	0 – 100	
Disease-oriented programs	1.1 HIV/AIDS and STIs	5.0	13.6	6.6	3.1	1.0	27.3	3
Non-disease-oriented programs	4.1 Health governance and financing; national health policies, strategies, and plans	8.3	13.4	7.1	4.1	1.1	38.4	1
	5.1 Alert and response capacities (for IHR)	6.2	14.4	7.6	3.1	1.1	37.7	2

a Abbreviations: HIV = human immunodeficiency virus; AIDS = acquired immunodeficiency syndrome; STI = sexually transmitted infection; IHR = International Health Regulations.

***Source***: Prepared by the authors from the study results.

The PAHO-adapted Hanlon method was approved by the PAHO Directing Council for implementation across the Region. To implement, PAHO first makes an official request to the national health authority of each Member State/Territory for one template (a fillable spreadsheet) with the results for that country. Each country template is based on national and, where appropriate, Regional considerations. PAHO supports a group approach for data collection, following the recommendations of Vilnius and Dandoy (10). In each country, a minimum of 6 national, government agency experts with broad knowledge of health and public health across a large spectrum of issues are invited to participate in a national prioritization session. The session takes about 2 – 4 hours using a spreadsheet.

### Limitations.

There were several limitations to this study. First, the participatory process assumed that the only actors with the capacity to contribute to developing the adapted method were among the representatives of the 12 Member States of the SPAG. This might have led to bias as academic institutions were excluded. However, PAHO invited academic professors to provide subject matter expertise at SPAG face-to-face meetings. In addition, several of the SPAG members were also university professors. Second, the range of points for the new components (inequity [E] and positioning [F]) was based on expert consensus and may need to be further adjusted and fine-tuned in the field. Third, there were issues with the operational definitions of some terms, e.g., effectiveness, coverage, and equity. Fourth, limited information for several programs and the subjective nature of criteria assessment may have led to inconsistent application of the methodology across countries in the Region. Fifth, lack of public health experts familiar with the national health situation and the work of PAHO may have been an issue.

### Conclusions

The PAHO-adapted Hanlon method has many advantages. First, the proposed new Hanlon method applies to both a "disease-oriented (pathogenic) vision” and a "health-oriented (salutogenic [15]) vision" (i.e., the non-disease-oriented vision). Second, it has a positive impact in the Region by focusing available PAHO resources on high priority programs. Third, the priority-setting process is transparent, objective, and applied in a systematic manner. Fourth, it is a participatory process that strengthens collaboration between PAHO and its Member States. Fifth, the PAHO-adapted Hanlon method has been validated against the ranking results from a more time consuming Delphi exercise among a group of senior health managers in Mexico, conducted by the Mexican Secretariat of Health independent of the SPAG process. Sixth, the proposed new method allows inclusion of important societal values, such as equity. Seventh, adjustment of the final priority score is enabled by an institutional positioning factor based on political consideration. This prevents the priority-setting exercise from becoming a purely mechanical process, and thereby increases its practical value in a real-world setting.

There are other methods for health-related decision making that involve scores, including the EVIDEM framework based on seven modules (16), and a Mapping of Multiple Criteria based on five categories and 31 criteria (17). Case examples of practical application of the Hanlon method for setting health priorities are available (18).

The PAHO-adapted Hanlon method has made a significant contribution to the science and practice of strategic planning. It has expanded the Hanlon method to encompass and align with new regional and global contexts by covering both disease- and non-disease-oriented programs and by considering equity and politics in priority-setting. The adapted method is more relevant and useful to the wider scope of health and public health. It will inform priority-setting as specified in the WHO 2016 Guide for Country Cooperation Strategy (19). The method may also be applicable to WHO, other WHO Regions, country governments, and other health institutions, all of which face similar needs in prioritizing both disease- and non-disease-oriented programs.

#### Author contributions.

BCKC, RAM, and OJM developed and implemented the project methodology. OJM and RAM with the PAHO Technical Team and PAHO SPAG (including BCKC) developed the initial PAHO adaptation of the Hanlon method and applied it with PAHO Member States. BCKC and RAM collected and responded to issues raised by the SPAG. OJM developed data collection and analysis tools for pilot testing. The PAHO Technical Team (including RAM and OJM) provided technical data required by the SPAG, and analytical support on pilot testing. SPAG members (including BCKC) collected issues from Member States and guided progress of the project. All authors contributed to the interpretation of results. BCKC wrote the first draft and RAM and OJM co-wrote the second draft of the manuscript. All authors contributed to subsequent revisions of various drafts, and final approval of the manuscript.

### Group authors.

***Members of the PAHO Strategic Plan Advisory Group who contributed as authors:*** Martha Leticia Caballero Abraham and Laura E. Gloria Hernández (Chairpersons), the Secretariat of Health, Mexico City, Mexico; Cristina Luna Ribadeneira and Peter N. Skerrett Guanoluisa (Vice Chairpersons), Ministry of Public Health, Quito, Ecuador; Keva S. Thompson, Ministry of Health, Nassau, Bahamas; Thaisa Santos Lima and Juliana V. Borges Vallini, Ministry of Health, Brasilia, Brazil; Bernard C.K. Choi, Public Health Agency of Canada, Ottawa, Ontario, Canada; Odette A. Urrutia Pumeyrau and Raquel Child Goldenberg, Ministry of Health, Santiago, Chile; María Rosibel Vargas Gamboa, Ministry of Health, San José, Costa Rica; Matías Villatoro, Nadia P.R. Villalta, and Maria Elena Marroquin, Ministry of Health, San Salvador, El Salvador; Michele Roofe and Karen Webster-Kerr, Ministry of Health, Kingston, Jamaica; Patricia A. Giménez León and Juan Carlos Coronel Zarate, Ministry of Public Health and Social Welfare, Asunción, Paraguay; Victor Raul Cuba Oré and Martin Yagui, Ministry of Health, Lima, Peru; and Jay McAuliffe, United States Centers for Disease Control and Prevention, Atlanta, Georgia, United States.

***Members of the PAHO Technical Team who contributed as authors:*** Federico Gerardo de Cosio, Blanca Cousiño, José A. Escamilla-Cejudo, Travis High, Rony A. Maza, Guillermo Mendoza, Andrea Morales, Oscar J. Mujica, Donna-Lisa Peña, Antonio Sanhueza, Maria Teresa Villen, and Daniel J. Walter, Pan American Health Organization, Regional Office of the World Health Organization for the Americas, Washington, DC, United States.

#### Acknowledgements.

The authors would like to express deep appreciation to PAHO for providing the webinar platform for the virtual meetings and travel funds for many members of the Strategic Plan Advisory Group attending face-to-face meetings. We gratefully acknowledge PAHO and the Secretariat of Health of Mexico for providing the meeting facility in Washington and Mexico City. Our gratitude to PAHO and Member States for their commitment and support of the project, and thanks to the external reviewers for their thoughtful and constructive suggestions on the manuscript.

#### Funding.

Funded by the Pan American Health Organization, Washington, DC, United States. The funders had no role in the study design, data collection or analysis, decision to publish, or preparation of the manuscript.

#### Disclaimer.

Authors hold sole responsibility for the views expressed in the manuscript, which may not necessarily reflect the opinion or policy of the *RPSP/PAJPH* and/or PAHO.

The views expressed in this paper are those of the authors and do not necessarily reflect those of the Public Health Agency of Canada (PHAC) and the Government of Canada.

The findings and conclusions in this report are those of the author(s) and do not necessarily represent the official position of the Centers for Disease Control and Prevention (CDC)/the Agency for Toxic Substances and Disease Registry.
